# The angiogenesis in decellularized scaffold-mediated the renal regeneration

**DOI:** 10.18632/oncotarget.7785

**Published:** 2016-02-27

**Authors:** Jin Mei, Yaling Yu, Miaozhong Li, Shanshan Xi, Sixiao Zhang, Xiaolin Liu, Junqun Jiang, Zhibin Wang, Jianse Zhang, Yuqiang Ding, Xinfa Lou, Maolin Tang

**Affiliations:** ^1^ Anatomy Department, Wenzhou Medical University, Wenzhou, 325035, China; ^2^ Institute of Bioscaffold Transplantation and Immunology, Wenzhou Medical University, Wenzhou, 325035, China; ^3^ Institute of Neuroscience, Wenzhou Medical University, Wenzhou, 325035, China; ^4^ Medical School of Ningbo University, Ningbo, 315211, China

**Keywords:** kidney regeneration, endothelial cells, endothelial progenitor cells, decellularized scaffolds, angiogenesis

## Abstract

There are increasing numbers of patients underwent partial nephrectomy, and recovery of disturbed renal function is imperative post partial nephrectomy. We previously have demonstrated the decellularized (DC) scaffolds could mediate the residual kidney regeneration and thus improve disturbed renal function after partial nephrectomy. However, the cellular changes including the angiogenesis in the implanted DC scaffold has not yet been elaborated. In this study, we observed that the scaffold promoted the proliferation of human umbilical vein endothelial cells (HUVEC) that adhered to the DC scaffold *in vitro*. We next examined the pathological changes of the implanted DC graft *in vivo*, and found a decreased volume of the scaffold and a dramatic angiogenesis within the scaffold. The average microvessel density (aMVD) increased at the early stage, while decreased at the later stage post transplantation. Expression level of vascular endothelial growth factor (VEGF) showed similar dynamic changes. In addition, many endothelial cells (ECs) and endothelial progenitor cells (EPCs) were distributed in the region which contained active angiogenesis in the scaffold. However, the implanted graft became fibrosis and the angiogenesis degraded at final stage roughly 8 weeks post transplantation. Our data indicate that DC scaffold can be vascularized *in vivo* and possible mechanisms are discussed.

## INTRODUCTION

Worldwide, renal cell carcinoma (RCC) ranks as the ninth most common cancer, there are increasingly numbers of patients underwent partial nephrectomy [1]. Despite kidney has relative functional compensation, some RCC cases might progress into chronic renal failure [2]. Patients living with unilateral kidney have relative higher risk of suffering chronic renal failure [3]. At present, the therapy strategies of chronic renal failure include high cost dialysis and kidney transplantation, which is always in short supply.

The tissue engineering seems to provide an alternative option for solving this problem. In recent years, the DC scaffold researches make remarkable progresses. Engineered tissues based on the DC tissues as a scaffold such as vessel [4], nerve [5], trachea [6, 7] and skin [8, 9] have been used in clinic. Most recently, the engineered solid organs such as heart [10], liver [11], lung [12, 13] and kidney [14, 15] have made breakthrough in preclinical research. The DC scaffolds have the following advantages. They have well biocompatibility beyond other biomaterials due to their natural origin, and can provide the native original three-dimensional structure and large numbers of cytokines for cell proliferation and differentiation. Additionally, residual proteins in DC scaffolds can regulate signal transduction pathways by modulating the activity of signalling molecules [16]. The above advantages have been identified by *in vitro* studies, but it remains poorly understood about effect of *in vivo* transplantation of DC scaffolds and possible mechanisms underlying DC scaffold-mediated organ regeneration.

Previously, we successfully found the regeneration of renal vessels in decellularized kidney post-implantation *in vivo* [17]. We transplanted the whole decellularized scaffold to the rat which received a left nephrectomy surgery. The protein markers (i.e. SMA and CD31) were regenerated, elastic fibers in vessels were intact and the thickness of vascular walls was increased post-surgery as time passed by. And we also made residual kidney regeneration by using DC kidney scaffold to repair partially resected kidney [18]. We implanted the scaffold *in vivo* onto the residual kidney of rats with partial resection, expecting the cells in residual kidney migrating into scaffold. Interestingly, the residual kidney continued to regenerate towards the distal end of the scaffold, and scaffold degraded gradually rather than renal cells migrated into the scaffold. The cellular mechanism underlying this phenomenon remains unknown and deserves further investigation. This study focuses on the cellular changes including the angiogenesis in the scaffold.

## RESULTS

### Implantation of DC kidney scaffold

**Figure 1:**
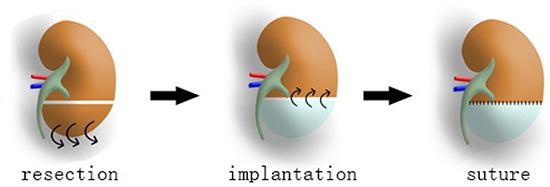
DC scaffold implantation into resected kidney *in vivo* First, lower 1/3 of the left renal parenchyma is transected. Then a similar size-scaffold (white part of the middle kidney) is grafted onto the cut-end by suture.

DC kidney scaffolds was prepared by detergent perfusion for implanting *in vivo*. Partial nephrectomy of the left kidney was performed. The lower 1/3 portion was removed, and then the similar size-DC scaffold was grafted onto the cut-end (Figure 1).

### Cellular pathologic changes in implanted DC kidney scaffold

**Figure 2:**
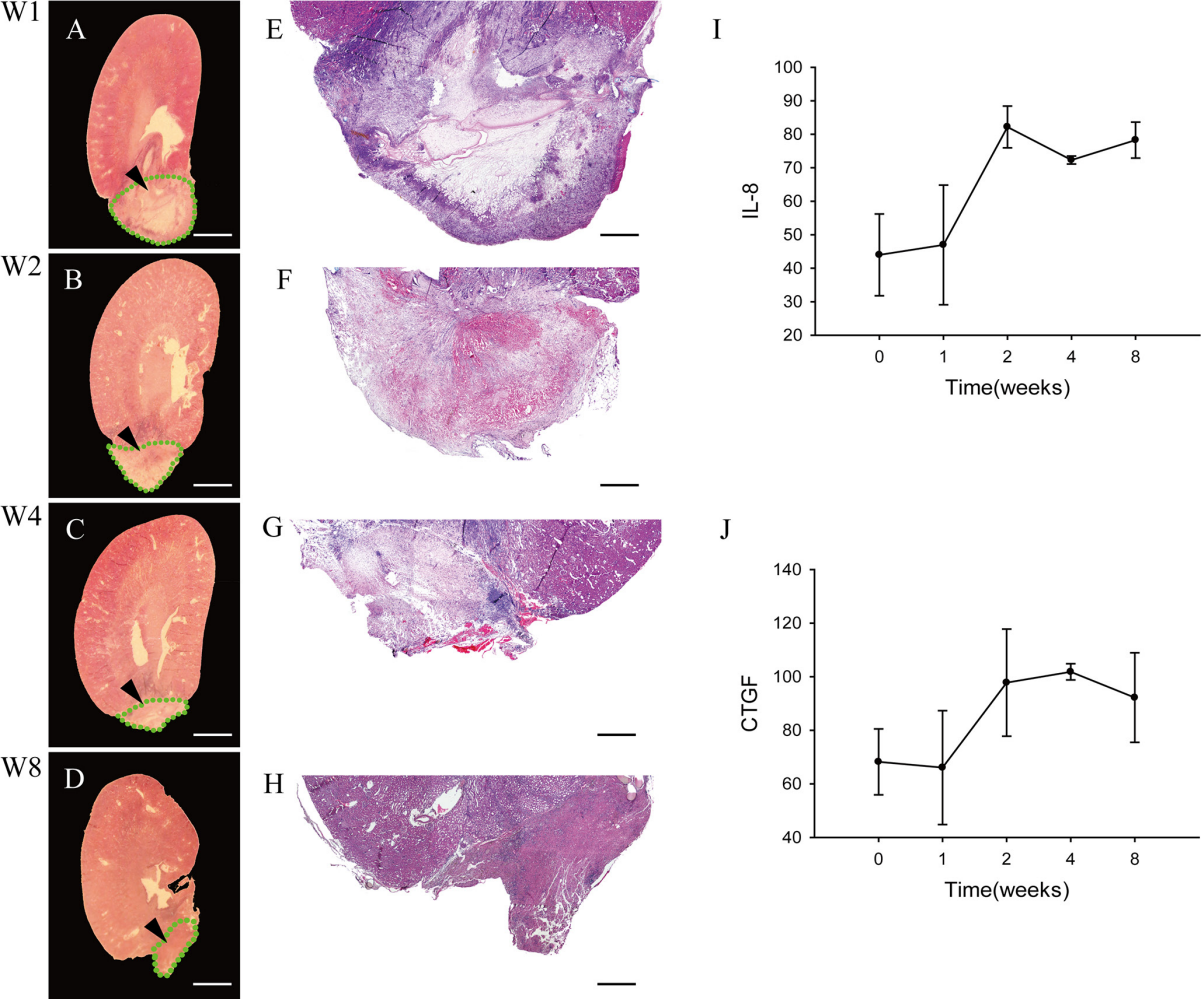
Pathologic changes of the implanted graft (**A–D**) Longitudinal cross-sections of whole experimental kidneys are observed under stereoscopic microscope on week 1 (A), 2 (B), 4 (C) and 8 (D). The structures that bold green dotted line indicate to are the residue of implanted DC kidney scaffolds. (**E–H**) Light microscopic examinations show the cellular pathologic results of the implanted grafts on week 1 (E), 2 (F), 4 (G) and 8 (H), respectively. On week 1, the outline of the implanted scaffold can be distinguished. The outer portion of the scaffold is infiltrated by massive inflammatory cells, while the inner part is not. On week 2, inflammatory cells also infiltrate deeply into the inner medulla. On week 4, the implanted graft becomes smaller, and the contour of inner and outer parts are not visible andinflammatory cells decrease also. On week 8, the implanted graft lost its original contour, with granulation tissue forming. (**I–J**) ELISA assay shows level of two cytokines IL-8 and CTGF in the implanted graft over time. Scale bar: A–D = 625 μm; E–H = 250 μm.

To examine cellular pathologic changes of the implanted graft in more detail, some rats were sacrificed on 1, 2, 4 and 8 weeks post-operation, and their kidneys were collected for analysis. As shown in Figure 2A–2H, the implanted graft became smaller in appearance over time. On week 1 and 2, the outline of the scaffold was relatively clear, as shown by the appearance of accumulated cells in the outer portion and less cells in the inner part of graft. These cells appeared to be inflammatory cells that infiltrated into the DC scaffold. Since week 4, the contour of the scaffold was still visible, but the difference between the outer and inner parts was not obvious, and on week 8 there were granulation-like tissue in the scaffold (Figure 2H).

Fibroblast is the main component cells of granulation tissue, and it synthesizes and secretes interleukin-8 (IL-8) [19]and connective tissue growth factor (CTGF) [20]. Then we performed ELISA assays to quantify levels of two cytokines, IL-8 and CTGF. The level of IL-8 increased and peaked at week 2 (Figure 2I), while CTGF was different. It reached to apex at week 4 and then began a slow decline (Figure 2J).

### Angiogenesis in implanted DC kidney scaffold

**Figure 3:**
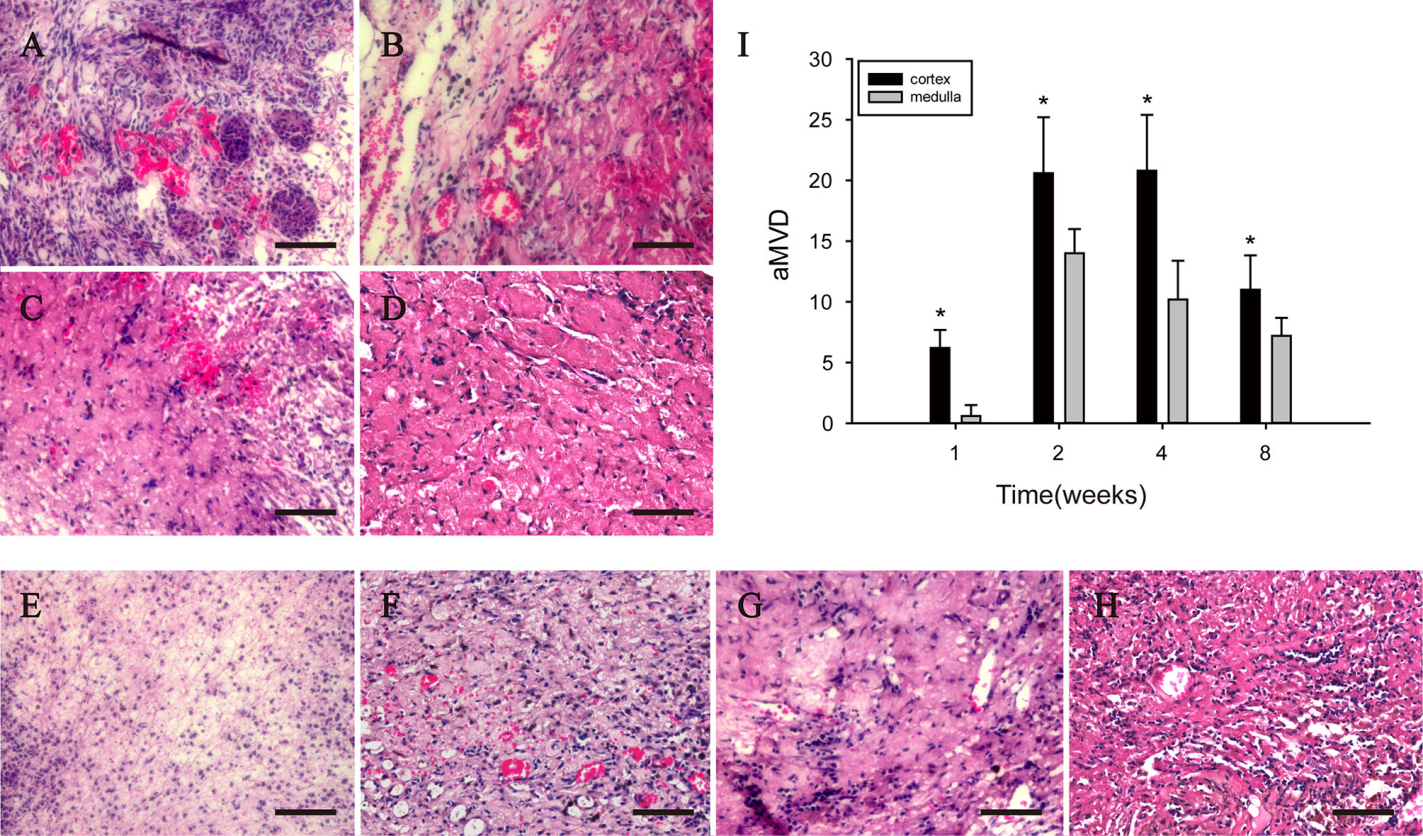
H & E staining shows that angiogenesis is present in the implanted scaffold (**A–D**) Tubular structures filling with red blood cells (arrows) are present in the outer part of the implanted scaffold. (**E–H**): Tubular structures filling with red blood cells (arrows) are present in the inner part. (**I**) shows the average microvessel density (aMVD) in the outer and inner parts.***p < 0.05. Scale bar: A–H = 50 μm.

H & E staining showed that many microvessels containing red blood cells were observed in the implanted DC kidney scaffold. On week 1, microvessels were present only in the outer part of the implanted scaffold (Figure 3A), and few of them were present in the inner part (Figure 3E). On week 2 and 4, microvessels were increased in number and distributed throughout the implanted scaffold (Figure 3B, 3C, 3F, 3G), but decreased dramatically on week 8 (Figure 3D, 3H, 3I).

**Figure 4:**
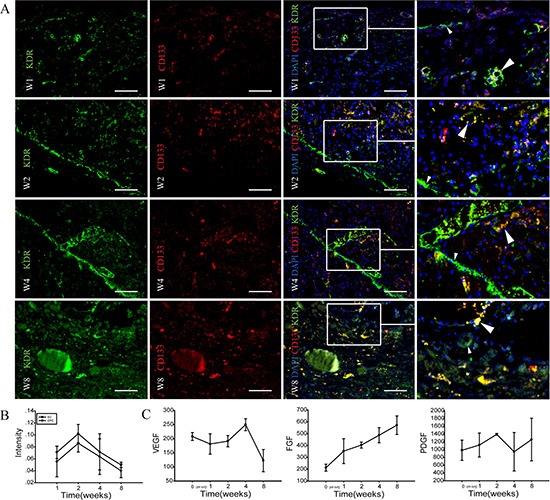
Immunohistochemical identification of angiogenesis in implanted-DC kidney scaffold (**A**) Immunofluorescence staining shows the stem cell mark CD133 (red), and the endothelial cell marker KDR (green) in implanted-DC kidney scaffold. White arrows point to KDR/CD133-colabeled cells. On week 1 (W1), scattered KDR^+^ cells and CD133^+^ cells are found. On week 2 (W2), plenty of KDR^+^ cells are distributed in a linear fashion (small arrow heads), while some KDR/CD133-colabeled cells are arranged in the wall of lumen-like structure (large arrow heads). On week 4 (W4), plenty of KDR^+^ cells are not only linearly distributed, but also arranged in the wall of blood vessel-like lumen. On week 8 (W8), KDR^+^ and CD133^+^ positive cells decrease. (**B**) Image-Pro plus analysis shows that the intensity of KDR^+^ and KDR^+^/CD133^+^ immunopositive cells increase at week 1, peak at week 2, and then decrease. The intensity of endothelial cells (KDR^+^ cells) are higher than the endothelial progenitor cells (bicolour positive-labeled cells). *p < 0.05. (**C**) Levels of three angiogenesis-related cytokines VEGF, FGF, PDGF in the implanted graft over time. Scale bar: A = 50 μm.

### Identification of angiogenesis in implanted DC kidney scaffold *in vivo*

Endothelial cells (EC) and endothelial progenitor cells (EPC) are involved in angiogenesis [21]. To further confirm the presence of angiogenesis in the implanted graft, we performed immunofluorescence staining to show EC with KDR, and EPC with KDR and CD133. On week 1, scattered KDR^+^ cells and CD133^+^ cells were found in the implanted scaffold. On week 2, plenty of KDR^+^ cells were distributed in a linear fashion, while some KDR^+^/CD133^+^-double positive cells were arranged in the wall of lumen-like structures. On week 4, plenty of KDR^+^ cells were not only linearly distributed, but also arranged in the inner wall of blood vessel-like lumen. KDR^+^ and CD133^+^ positive cells were scattered. On week 8, KDR^+^ and CD133^+^ positive cells decreased. Image-Pro plus analysis showed that the intensity of angiogenesis-related cells (mainly EC) increased at first week, peaked at week 2, and then decreased.

VEGF is a major contributor in angiogenesis, by increasing the number of capillaries in a given network [22] and FGF stimulates the proliferation of fibroblasts and endothelial cells which give rise to angiogenesis [23]. PDGF recruits smooth muscle cells during angiogenesis [24]. To know possible mechanism underlying the angiogenesis, we measured the levels of VEGF, FGF and PDGF in implanted grafts by ELISA assays. VEGF increased at week 4, but then decreased. FGF was increasing during the 8-week period after transplanting *in vivo*. PDGF was roughly unchanged.

### Proliferation of HUVECs in seeding on DC scaffolds *in vitro*

**Figure 5:**
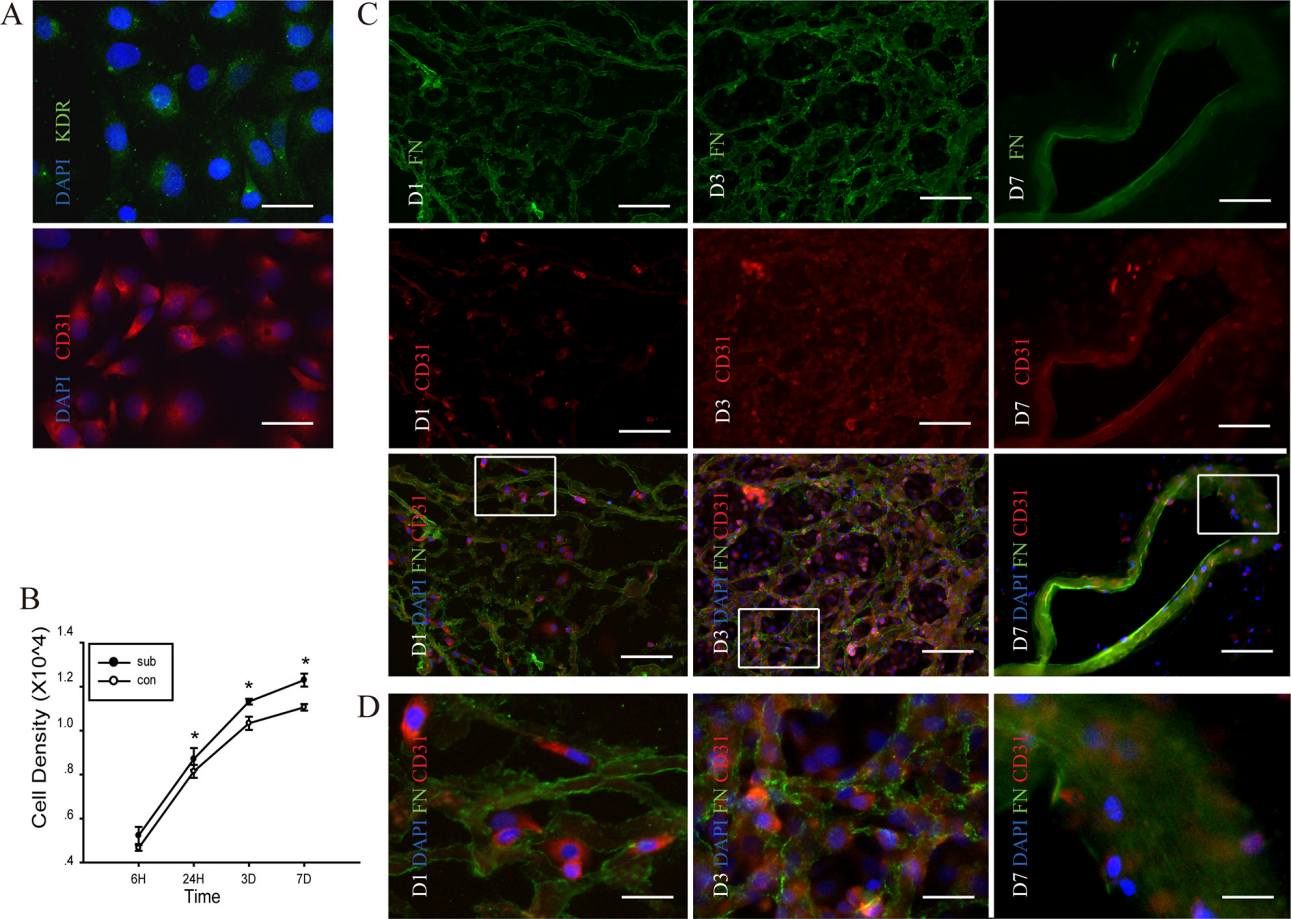
Proliferation of human umbilical vein endothelial cells (HUVEC) in the DC kidney scaffolds *in vitro* (**A**) Immunofluorescence identification of KDR (green) and CD31 (red) in HUVEC. (**B**) CCK-8 proliferation assay shows that comparing with those without scaffolds, cells seeding on the scaffolds proliferate highly at day 1, 3 and 7 after seeding on the scaffold. There is statistical difference in proliferate ability between cells with and without DC scaffolds. (**C**) Double immunofluorescence shows the scaffold and the HUVEC with fibronectin (green) and CD31 (red), respectively. On the first day after seeding on the scaffold, a small number of HUVECs adheres to the scaffold. On the third day, adhered HUVECs are increased. On the seventh day, HUVECs adhere to the wall of median renal vessel-like structure in the scaffolds. (**D**) The magnification pictures show the white squares in Figure. C. *p < 0.05 Scale bar: A = 12.5 μm, C =50 μm D = 12.5 μm.

HUVECs were used as a laboratory model system for the study of angiogenesis [25]. As shown in Figure 5A, the two antigens KDR and CD31 were used to identify HUVECs, and both two antigens were located on the cell membrane. They can proliferate and produce new extracellular (Figure 5C). CCK-8 proliferation assay showed that after seeding on the DC scaffold, proliferate ability was slight higher at day 1but more frequently at day 3 and 7, compared with those without scaffold (Figure 5B). In addition, HUVEC adhered to the kidney scaffold was also increased over time, as shown by the enhanced adhesion rate (Figure 5B). On the first day after seeding on the scaffold, a small number of HUVECs adhered to the scaffold and adhered HUVECs were increased on the third day. On the seventh day, HUVECs adhered to the wall of median renal vessel-like structures in the scaffolds.

## DISCUSSION

The success of residual kidney regeneration in above model has been reported in our previous study, as shown by functional recovery and data of macroscopic examination [18]. The cellular events in the regenerated renal tissue is still poorly understood. In this study, we have shown that there are dynamic processes of angiogenesis and fibrosis in the implanted scaffold, and these results are helpful for understanding the role of the DC kidney scaffold in the renal regeneration.

Previously, vascularized sheets have been used to promote tissue regeneration in heterotopic transplantation model such as in subcutaneous region [26, 27] and peritoneum [28]. In our previously reported orthotopic implantation model, residual kidney continued to regenerate towards the graft end. And in this paper, we observed the scaffold it self was vascularised. Much richer blood supply in the kidney than in subcutaneous and peritoneum may be a key factor for angiogenesis after the transplantation.

In our experimental model, the implanted scaffold became smaller overtime and angiogenesis occurred in the scaffold. AMVD in the implanted scaffold increased explosively 4 weeks post implantation. We found that angiogenesis-related growth factors (i.e. VEGF, PDGF, FGF) displayed different dynamic changes within the implanted scaffold. VEGF level peaked in week 4but sharply declined in week 8. WhilePDGF remained unchanged but PDGF kept increasing trend after transplanting *in vivo*. It is likely that the scaffold may trigger or promote the angiogenesis by releasing some cytokines such as VEGF, FGF and PDGF. This idea is supported by the data from the increased proliferation of HUVECs seeded in DC kidney scaffold *in vitro*. Previous studies have shown that the regeneration of the organ depends on the angiogenesis and paracrine of cytokines from angiogenesis stimulates the regeneration of adjacent tissues [29, 30]. Taken together, it might be possible that the DC kidney scaffold releases some angiogenic cytokines, which promote the proliferation of endothelial cells and contribute to the angiogenesis in the scaffold. Meanwhile, the cytokines secreted from the angiogenesis stimulates the regeneration of residual kidney into the scaffold. Further studies are needed to explore this possibility in detail.

Previous study revealed that degradation of extracellular matrix is exaggerated after *in vivo* implantation, thus resulting in impairment of the migration of endothelial cells and inhibition of angiogenesis [31]. Consistently, we observed that the scaffold became smaller during a 8-week period after transplantation, and aMVD within the graft was dramatic decreased, which was accompanied by the reduction of the intensity of ECs and EPCs. On the other hand, we found that the implanted scaffold became granulation tissue, with increasing CTGF, FGF and PDGF. It can be evolved that inflammatory cells proliferation and naturally degradation of implanted scaffold reduces angiogenesis.

Our results demonstrate that the DC kidney scaffolds can be vascularised. *In vitro* HUVEC can adhere to the renal vessels of DC kidney scaffold, and the proliferative ability was enhanced. *In vivo*, the aMVD within the scaffold increased first, but then decreased. Expression level of VEGF showed similar dynamic changes. In addition, ECs and EPCs were distributed in the region which contained active angiogenesis in the scaffold. However, the implanted graft became fibrosis and the angiogenesis degraded at final stage roughly 8 weeks post transplantation. Normally, the extracellular matrix secreted by cells is degraded by cells, the possible mechanism were reported until very recently. [32, 33] But the degraded mechanism of implanted scaffolds has not been mentioned in the published literatures. The DC scaffold-mediated injured kidney regeneration *in vivo* may be achieved by release angiogenesis-related growth factors to promote the proliferation of ECs and EPCs, which contribute to the angiogenesis and kidney regeneration.

## MATERIALS AND METHODS

### 1.1 Animals and DC kidney scaffolds

Sprague Dawley (SD) rats at the age of about two months, weighing about 250 g were obtained from the Laboratory Animal Center of Wenzhou Medical University, Zhejiang Province, China. The experiments were conducted in accordance with ethical guidelines for the use and care of animals and the study was approved by the administration of Wenzhou Medical University.

Our method for preparation of DC kidney scaffolds was previously described [18] Briefly, solutions were perfused through the infrarenal abdominal aorta at an approximate rate of 8 ml/min in the following order: 50 U/ml heparin in 0.01 M phosphate buffered saline (PBS) for 30 min, 0.1% triton X-100 for 3 h, deionized water for 30 min, 0.8% (v/v) sodium lauryl sulfate (SDS) for 3 h, and deionized-water containing 100 U/ml penicillin and 100 μg/ml streptomycin for 24 h.

### 1.2 Transplantation of DC kidney scaffolds

Animals were anesthetized with an intraperitoneal injection of chloral hydrate (6 ml/kg body weight), and an abdominal paramedian incision was made from the pubis to the xyphoid to expose the left kidney. In the beginning of the operation, 1 ml of heparin (50 U/ml) was injected through the inferior vena cava. After separating the left renal capsule, renal artery and vein were clipped with micro-ligation haemostatic clip (W40130; Chen-He Microsurgical Instruments Factory, China), at which point the timer was started to make sure renal ischemia was kept under 10 min. The left kidney was transected slightly below the renal pelvis (removing about 1/3 of the renal parenchyma). The wound was grafted with lower 1/3 of DC scaffold by suturing the external capsules between the excised kidney and DC kidney scaffold. After reperfusion, the left kidney was monitored if there was a leakage of blood for 20 min before closing the abdominal wall. After surgery, all animals were given unlimited access to rat chow and water containing penicillin and streptomycin.

### 1.3 Enzyme-like immune sorbent assay (ELISA) for quantitative analysis of cytokines

Total proteins in the implanted scaffolds were extracted using an ELISA kit (R & D Systems, USA). The concentrations of cytokines including VEGF, IL-8, PDGF, FGF and CTGF were measured with a microplate reader at 450 nm [34].

### 1.4 Average microvessel density

Average microvessel density (aMVD) in the DC scaffold implants was assessed through the hematoxylin-eosin (H & E) staining according to the method described by Weidner et al. [35] First, H & E stained renal tissues in the implanted scaffold were screened at low magnification (100x) to identify hot spots of the angiogenesis. Within hot-spot areas, the microvessels were counted in a single high-power (200x) field, and the average vessel counted in three hot spots was considered as the value of aMVD. All counts were performed by three investigators in a blinded manner. Comparisons regarding microvessel counts were drawn among the observers and discrepant results were reassessed. The consensus was used as the final score for analysis.

### 1.5 Human umbilical vein endothelial cells seeding in DC kidney scaffolds

Human umbilical vein endothelial cells (HUVEC) were obtained from PromoCell (Heidelberg, Germany) and were cultured in DMEM medium supplemented with 10% FBS at 37°C in a humidified atmosphere containing 50% CO_2_. For the studies, only cells at passage 3–5 were used. DC kidney scaffolds were cut into 100 μm-thick sections and sticked to 12 mm cover slips (Hong- da Medical Equipment Co., China). Before seeding cells, scaffold-coated cover slips and non-coated cover slips were washed three times by PBS, and then were immersed with penicillin-streptomycin solution (P1400, Solarbio Science & Technology Co., China) overnight. After removed the antibiotics, HUVEC were seeded at 20,000 cells/cm^2^ on all cover slips in 24 well plates.

### 1.6 Cell proliferation assay

Cell proliferation was measured by Cell-Counting kit-8 (CCK-8) (Beyotime, Shanghai, China) according to the manufacturer's instructions. Cells were incubated in 10% CCK-8 that was diluted in normal culture medium at 37°C until the visual colour conversion occurred. Proliferation rates were determined at 6, 24, 72 and 148 hours after seeding in DC kidney scaffolds.

### 1.7 Histology and immunofluorescence analysis

Samples were prepared for histological and immunofluorescence analyses by following standard protocols for paraffin embedding. For histological analysis, mounted kidney sections were stained by hematoxylin and eosin.

For immunofluorescence staining, the sections were blocked with 5% normal bovine serum in PBS for 30 min. Sections were then incubated with primary antibodies overnight at 4°C. The primary antibodies used were as follows: mouse anti-VEGFR2 (1:200; Abcam, UK), rabbitanti-CD133 (1:200; Abcam), goat anti-fibronectin (1:200; Abcam) and rabbit anti-CD31 (1:200; Abcam). After being washed in PBS, sections were then incubated in 488- or 594-conjugated species-specific secondary antibody (1:100; Chemicon, USA) for 2 hours. Slides were observed with an Olympus fluorescent microscope and images were captured using Olympus soft image viewer. The intensity of immunoreactivity was quantified using the Image-Pro Plus 6.0 software (Media Cybernetics, USA).

### 1.8 Statistical analysis

SPSS 17.0 (SPSS Inc., Chicago, USA) was used for statistical analysis. Statistical data were reported as mean ± standard deviation. Independent samples *t*-test was used to compare the levels of different experimental groups. *P*-Values of < 0.05 were considered statistically significant.

## References

[1] Jonasch E, Gao J, Rathmell WK (2014). Renal cell carcinoma. Bmj.

[2] Venkatachalam MA, Griffin KA, Lan R, Geng H, Saikumar P, Bidani AK (2010). Acute kidney injury: a springboard for progression in chronic kidney disease. Am J Physiol Renal Physiol.

[3] Lankadeva YR, Singh RR, Tare M, Moritz KM, Denton KM (2014). Loss of a kidney during fetal life: long-term consequences and lessons learned. Am J Physiol Renal Physiol.

[4] Chemla ES, Morsy M (2009). Randomized clinical trial comparing decellularized bovine ureter with expanded polytetrafluoroethylene for vascular access. The British journal of surgery.

[5] Karabekmez FE, Duymaz A, Moran SL (2009). Early clinical outcomes with the use of decellularized nerve allograft for repair of sensory defects within the hand. Hand (NY).

[6] Gonfiotti A, Jaus MO, Barale D, Baiguera S, Comin C, Lavorini F, Fontana G, Sibila O, Rombola G, Jungebluth P, Macchiarini P (2014). The first tissue-engineered airway transplantation: 5-year follow-up results. Lancet.

[7] Macchiarini P, Jungebluth P, Go T, Asnaghi MA, Rees LE, Cogan TA, Dodson A, Martorell J, Bellini S, Parnigotto PP, Dickinson SC, Hollander AP, Mantero S (2008). Clinical transplantation of a tissue-engineered airway. Lancet.

[8] Butler CE, Langstein HN, Kronowitz SJ (2005). Pelvic, abdominal, and chest wall reconstruction with AlloDerm in patients at increased risk for mesh-related complications. Plastic and reconstructive surgery.

[9] Albo D, Awad SS, Berger DH, Bellows CF (2006). Decellularized human cadaveric dermis provides a safe alternative for primary inguinal hernia repair in contaminated surgical fields. American journal of surgery.

[10] Ott HC, Matthiesen TS, Goh SK, Black LD, Kren SM, Netoff TI, Taylor DA (2008). Perfusion-decellularized matrix: using nature's platform to engineer a bioartificial heart. Nature medicine.

[11] Uygun BE, Soto-Gutierrez A, Yagi H, Izamis ML, Guzzardi MA, Shulman C, Milwid J, Kobayashi N, Tilles A, Berthiaume F, Hertl M, Nahmias Y, Yarmush ML (2010). Organ reengineering through development of a transplantable recellularized liver graft using decellularized liver matrix. Nature medicine.

[12] Ott HC, Clippinger B, Conrad C, Schuetz C, Pomerantseva I, Ikonomou L, Kotton D, Vacanti JP (2010). Regeneration and orthotopic transplantation of a bioartificial lung. Nature medicine.

[13] Petersen TH, Calle EA, Zhao L, Lee EJ, Gui L, Raredon MB, Gavrilov K, Yi T, Zhuang ZW, Breuer C, Herzog E, Niklason LE (2010). Tissue-engineered lungs for *in vivo* implantation. Science.

[14] Song JJ, Guyette JP, Gilpin SE, Gonzalez G, Vacanti JP, Ott HC (2013). Regeneration and experimental orthotopic transplantation of a bioengineered kidney. Nature medicine.

[15] Ross EA, Williams MJ, Hamazaki T, Terada N, Clapp WL, Adin C, Ellison GW, Jorgensen M, Batich CD (2009). Embryonic stem cells proliferate and differentiate when seeded into kidney scaffolds. Journal of the American Society of Nephrology.

[16] Lelongt B, Ronco P (2003). Role of extracellular matrix in kidney development and repair. Pediatric nephrology.

[17] Zhang J, Wang Z, Lin K, Yu Y, Zhao L, Chu T, Wu L, Alkhawaji A, Li M, Shao Y, Li T, Lou X, Chen S (2015). *In vivo* regeneration of renal vessels post whole decellularized kidneys transplantation. Oncotarget.

[18] Yu YL, Shao YK, Ding YQ, Lin KZ, Chen B, Zhang HZ, Zhao LN, Wang ZB, Zhang JS, Tang ML, Mei J (2014). Decellularized kidney scaffold-mediated renal regeneration. Biomaterials.

[19] Vlahopoulos S, Boldogh I, Casola A, Brasier AR (1999). Nuclear factor-kappaB-dependent induction of interleukin-8 gene expression by tumor necrosis factor alpha: evidence for an antioxidant sensitive activating pathway distinct from nuclear translocation. Blood.

[20] Jun JI, Lau LF (2011). Taking aim at the extracellular matrix: CCN proteins as emerging therapeutic targets. Nature reviews Drug discovery.

[21] Bobryshev YV, Orekhov AN, Chistiakov DA (2015). Vascular stem/progenitor cells: current status of the problem. Cell and tissue research.

[22] Goto F, Goto K, Weindel K, Folkman J (1993). Synergistic effects of vascular endothelial growth factor and basic fibroblast growth factor on the proliferation and cord formation of bovine capillary endothelial cells within collagen gels. Laboratory investigation; a journal of technical methods and pathology.

[23] Ornitz DM, Itoh N (2001). Fibroblast growth factors. Genome biology.

[24] Hoch RV, Soriano P (2003). Roles of PDGF in animal development. Development.

[25] Park HJ, Zhang Y, Georgescu SP, Johnson KL, Kong D, Galper JB (2006). Human umbilical vein endothelial cells and human dermal microvascular endothelial cells offer new insights into the relationship between lipid metabolism and angiogenesis. Stem cell reviews.

[26] Pileggi A, Molano RD, Ricordi C, Zahr E, Collins J, Valdes R, Inverardi L (2006). Reversal of diabetes by pancreatic islet transplantation into a subcutaneous, neovascularized device. Transplantation.

[27] Fox IJ, Schafer DF, Yannam GR (2006). Finding a home for cell transplants: location, location, location. Am J Transplant.

[28] Yokoo T, Matsumoto K, Yokote S (2011). Potential use of stem cells for kidney regeneration. International journal of nephrology.

[29] Ding BS, Nolan DJ, Guo P, Babazadeh AO, Cao Z, Rosenwaks Z, Crystal RG, Simons M, Sato TN, Worgall S, Shido K, Rabbany SY (2011). Endothelial-derived angiocrine signals induce and sustain regenerative lung alveolarization. Cell.

[30] Ding BS, Nolan DJ, Butler JM, James D, Babazadeh AO, Rosenwaks Z, Mittal V, Kobayashi H, Shido K, Lyden D, Sato TN, Rabbany SY, Rafii S (2010). Inductive angiocrine signals from sinusoidal endothelium are required for liver regeneration. Nature.

[31] Elpek GO (2015). Angiogenesis and liver fibrosis. World journal of hepatology.

[32] Ruggiero C, Fragassi G, Grossi M, Picciani B, Di Martino R, Capitani M, Buccione R, Luini A, Sallese M (2015). A Golgi-based KDELR-dependent signalling pathway controls extracellular matrix degradation. Oncotarget.

[33] Albeiroti S, Ayasoufi K, Hill DR, Shen B, de la Motte CA (2015). Platelet hyaluronidase-2: an enzyme that translocates to the surface upon activation to function in extracellular matrix degradation. Blood.

[34] Engvall E, Perlmann P (1971). Enzyme-linked immunosorbent assay (ELISA). Quantitative assay of immunoglobulin G. Immunochemistry.

[35] Weidner N, Semple JP, Welch WR, Folkman J (1991). Tumor angiogenesis and metastasis- correlation in invasive breast carcinoma. New England Journal of Medicine.

